# Supplementary material for: Mitochondrial ABHD11 inhibition drives sterol metabolism to modulate T-cell effector function
Source: Nat Commun. 2025 Nov 3;16:9484. doi: 10.1038/s41467-025-65417-4 (PMC12583646; doi:10.1038/s41467-025-65417-4)

SUPPLEMENTARY INFORMATION

Title: Mitochondrial ABHD11 inhibition drives sterol metabolism to modulate T cell effector function

Authors: Benjamin J. Jenkins[1,†], Yasmin R. Jenkins[1,†], Fernando M. Ponce-Garcia[1], Chloe Moscrop[2], Iain A. Perry[1], Matthew D. Hitchings[1], Alejandro H. Uribe[3], Federico Bernuzzi[3], Simon Eastham[2], James G. Cronin[1], Ardena Berisha[4], Alexandra Howell[5], Joanne Davies[5], Julianna Blagih[6,7], Marta Williams[8], Morgan Marsden[8], Douglas J. Veale[9], Luke C. Davies[1], Micah Niphakis[10], David K. Finlay[11], Linda V. Sinclair[12], Benjamin F. Cravatt[10], Andrew E. Hogan[4], James A. Nathan[13], Ian R. Humphreys[8], Ursula Fearon[14], David Sumpton[3], Johan Vande Voorde[3,15], Goncalo Dias do Vale[16], Jeffrey G. McDonald[16], Gareth W. Jones[2], James A. Pearson[5,‡], Emma E. Vincent[17,18,‡], Nicholas Jones[1,‡,*]

[1] Institute of Life Science, Swansea University Medical School, Swansea University, SA2 8PP, UK.
[2] Cellular and Molecular Medicine, University of Bristol, Biomedical Sciences Building, Bristol, BS8 1TD, UK.
[3] Cancer Research UK Scotland Institute, Garscube Estate, Switchback Road, Glasgow, G61 1BD, UK.
[4] Kathleen Lonsdale Institute for Human Health Research, Maynooth University, Maynooth, Co. Kildare, Ireland
[5] Diabetes Research Group, Division of Infection and Immunity, School of Medicine, Cardiff University, CF14 4XN, UK
[6] The Francis Crick Institute, 1 Midland Road, London, NW1 1AT, UK.
[7] University of Montreal, Maisonneuve-Rosemont Hospital Research Centre, Montreal, 5414 Assomption Blvd, H1T 2M4, Canada
[8] Division of Infection and Immunity/Systems Immunity University Research Institute, School of Medicine, Cardiff University, Cardiff, CF14 4XN, UK.
[9] EULAR Centre of Excellence, Centre for Arthritis and Rheumatic Diseases, St Vincent's University Hospital, Dublin, Ireland.
[10] Department of Chemistry, Scripps Research, La Jolla, California 92037, United States
[11] School of Biochemistry and Immunology, Trinity Biomedical Sciences Institute, Trinity College Dublin, 152-160 Pearce Street, Dublin, Ireland
[12] Division of Cell Signalling and Immunology, School of Life Sciences, University of Dundee, Dundee, UK
[13] Cambridge Institute of Therapeutic Immunology & Infectious Disease (CITIID), Jeffrey Cheah Biomedical Centre, Department of Medicine, University of Cambridge, Cambridge, CB2 0AW, UK.
[14] Molecular Rheumatology, School of Medicine, Trinity Biomedical Sciences Institute, Trinity College Dublin, 152-160 Pearce Street, Dublin, Ireland.
[15] School of Cancer Sciences, Wolfson Wohl Cancer Research Centre, University of Glasgow, Glasgow, G61 1QH, UK
[16] Center for Human Nutrition, Department of Molecular Genetics, University of Texas Southwestern Medical Center, Dallas, United States.
[17] School of Translational Health Sciences, Dorothy Hodgkin Building, University of Bristol, Bristol, BS1 3NY, UK.
[18] Integrative Epidemiology Unit, School of Population Health Science, University of Bristol, Bristol, BS8 2BN, UK.

[†] These authors contributed equally
[‡] These authors jointly supervised this work
[*] Corresponding author: Nicholas Jones, Institute of Life Science, Swansea University Medical School, Swansea, SA2 8PP, UK, +44 (0)1792 513509, n.jones@swansea.ac.uk

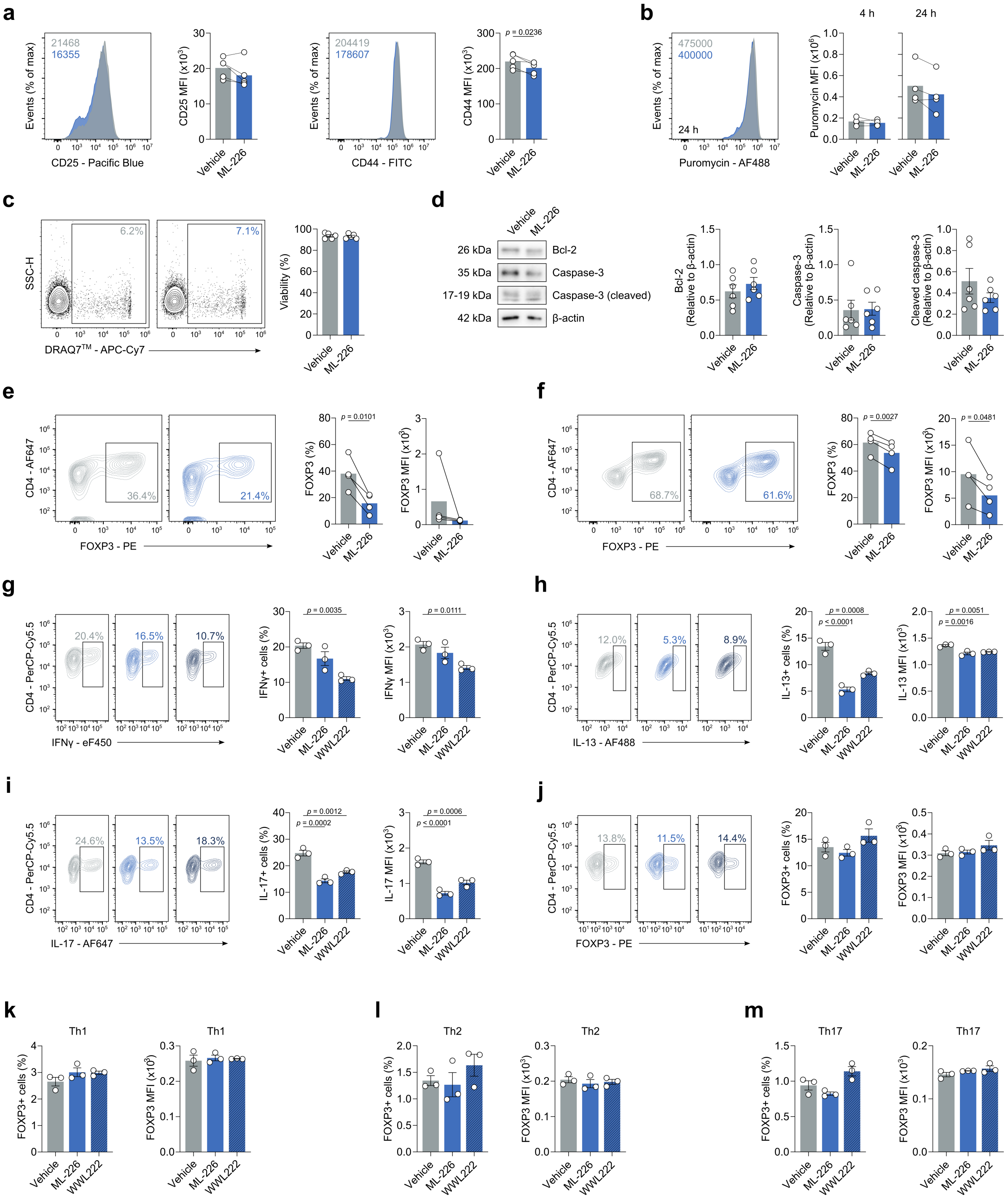

Supplementary Figure 1. AHBD11 inhibition does not significantly alter T-cell size, protein translation and viability

(Figure legend on next page)

Supplementary Figure 1. AHBD11 inhibition does not significantly alter T-cell size, protein translation and viability

(**a**) Surface expression of activation markers (CD25 and CD44) on CD4+ effector T-cells (n = 5). (**b**) Puromycin incorporation in CD4+ effector T-cells (n = 4). (**c**) Cell viability, as determined by DRAQ7®, in CD4+ effector T-cells (n = 5). (**d**) Bcl-2 and caspase-3 expression in CD4+ T-cells (n = 6). Protein loading assessed using β-actin. (**e**) Intracellular FOXP3 expression in CD4+ naïve T-cells following polarisation towards Treg cells in the presence and absence of ML-226 (n = 4). (**f**) Intracellular FOXP3 expression in already-polarised CD4+ Treg cells activated the presence and absence of ML-226 (n = 4). (**g**) Intracellular IFNγ expression in murine CD4+ effector T-cells following polarisation towards Th1 in the presence and absence of ML-226 or WWL222 (n = 3). (**h**) Intracellular IL-13 expression in murine CD4+ effector T-cells following polarisation towards Th2 in the presence and absence of ML-226 or WWL222 (n = 3). (**i**) Intracellular IL-17 expression in murine CD4+ effector T-cells following polarisation towards Th17 in the presence and absence of ML-226 or WWL222 (n = 3). (**j**) Intracellular FOXP3 expression in murine CD4+ effector T-cells following polarisation towards Treg in the presence and absence of ML-226 or WWL222 (n = 3). (**k**) Intracellular FOXP3 expression in murine CD4+ effector T-cells following polarisation towards Th1 in the presence and absence of ML-226 or WWL222 (n = 3). (**l**) Intracellular FOXP3 expression in murine CD4+ effector T-cells following polarisation towards Th2 in the presence and absence of ML-226 or WWL222 (n = 3). (**m**) Intracellular FOXP3 expression in murine CD4+ effector T-cells following polarisation towards Th17 in the presence and absence of ML-226 or WWL222 (n = 3). All experiments were carried out using human samples, unless otherwise stated. CD4+ T-cells were activated with α-CD3 (2 μg/ml) and α-CD28 (20 μg/ml) for 24 h, in the presence and absence of ML-226, unless otherwise stated. Data are expressed as either: mean, with paired dots representing biological replicates; or mean ± SEM. Statistical tests used: two-tailed paired T-test (**a**, **b**, **e**, **f**), two-tailed unpaired T-test (**c**, **d**), one-way ANOVA with Dunnett's multiple comparisons test (**g-m**). Source data are provided as a Source Data file.

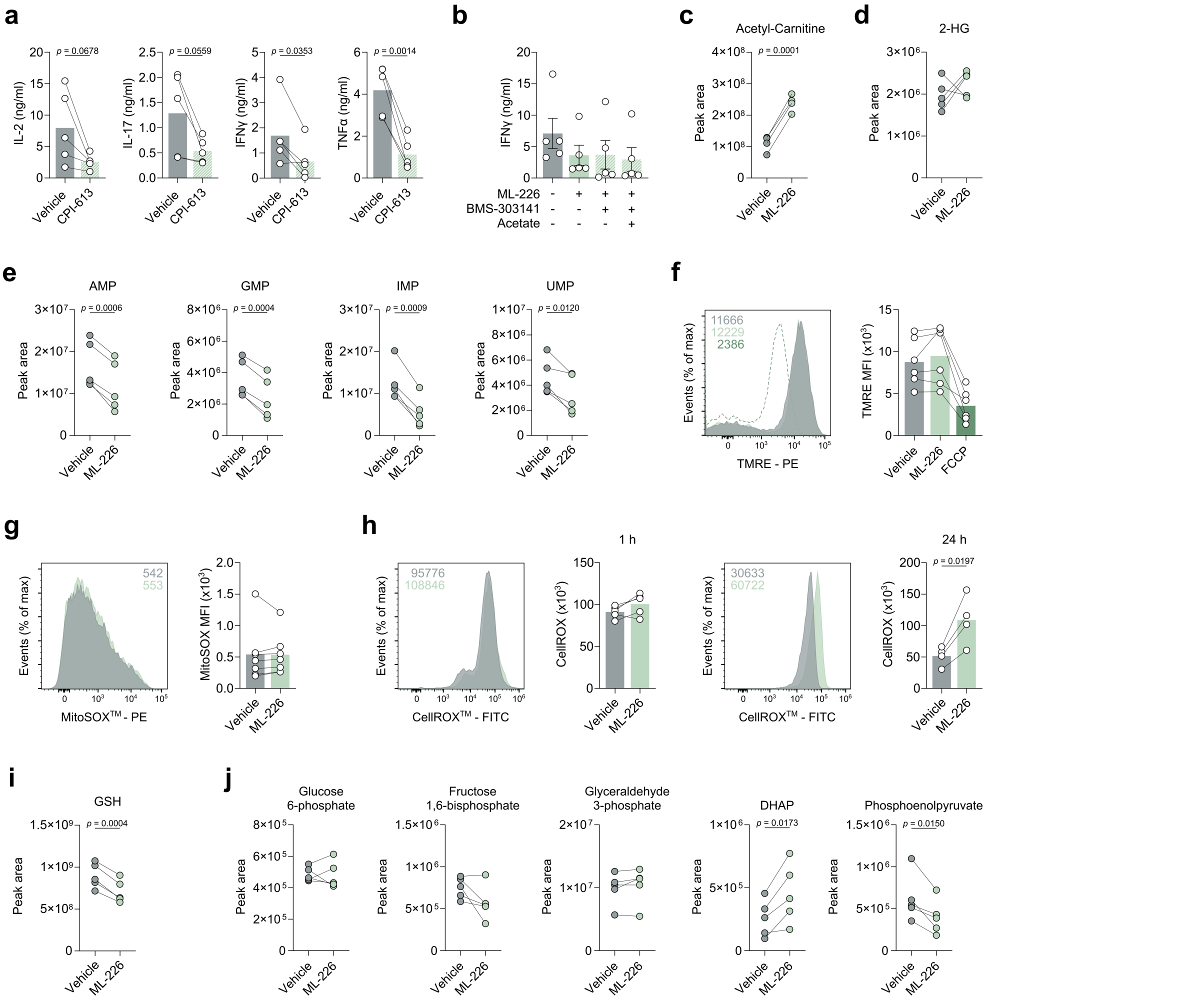

Supplementary Figure 2. ABHD11 inhibition reduces intracellular monophosphate nucleotides

(Figure legend on next page)

Supplementary Figure 2. ABHD11 inhibition reduces intracellular monophosphate nucleotides

(**a**) IL-2, IL-17, IFNγ and TNFα production by CD4+ effector T-cells treated with CPI-613 (n = 5). (**b**) IFNγ production by CD4+ T-cells, activated in the presence and absence of ML-226 and BMS-303141 (n = 5). (**c**) Intracellular levels of acetyl-carnitine in CD4+ T effector cells (n = 4). (**d**) Intracellular levels of 2-hydroxyglutarate (2-HG) in CD4+ T effector cells (n = 5). (**e**) Intracellular levels of selected monophosphate nucleotides in CD4+ effector T-cells. Metabolites include: inosine monophosphate, adenosine monophosphate, guanosine monophosphate and uridine monophosphate (n = 5). (**f**) Mitochondrial membrane potential, as determined by TMRE staining, in CD4+ effector T-cells (n = 6). FCCP (1 μM) was used as a positive control. (**g**) Mitochondrial reactive oxygen species (ROS) levels, as determined by MitoSOX™ Red, in CD4+ effector T-cells at 24 h (n = 7). (**h**) Total ROS levels, as determined by CellROX™ Green, in CD4+ T-cells (1 h: n = 5; 24 h: n = 4). (**i**) Intracellular levels of glutathione in CD4+ effector T-cells (n = 5). (**j**) Intracellular levels of selected glycolytic intermediates in CD4+ effector T-cells. Metabolites include: glucose 6-phosphate, fructose 1,6-bisphosphate, glyceraldehyde 3-phosphate, dihydroxyacetone phosphate (DHAP) and phosphoenolpyruvate (n = 5). All experiments were carried out using human samples. CD4+ T-cells were activated with α-CD3 (2 μg/ml) and α-CD28 (20 μg/ml) for 24 h, in the presence and absence of ML-226, unless otherwise stated. Data are expressed as either: mean, with paired dots representing biological replicates; or mean ± SEM. Statistical tests used: two-tailed paired T-test (**a**, **c**-**e**, **g**-**j**), one-way ANOVA with Dunnett's multiple comparisons test (**b**), repeated-measures one-way ANOVA (**f**). Source data are provided as a Source Data file.

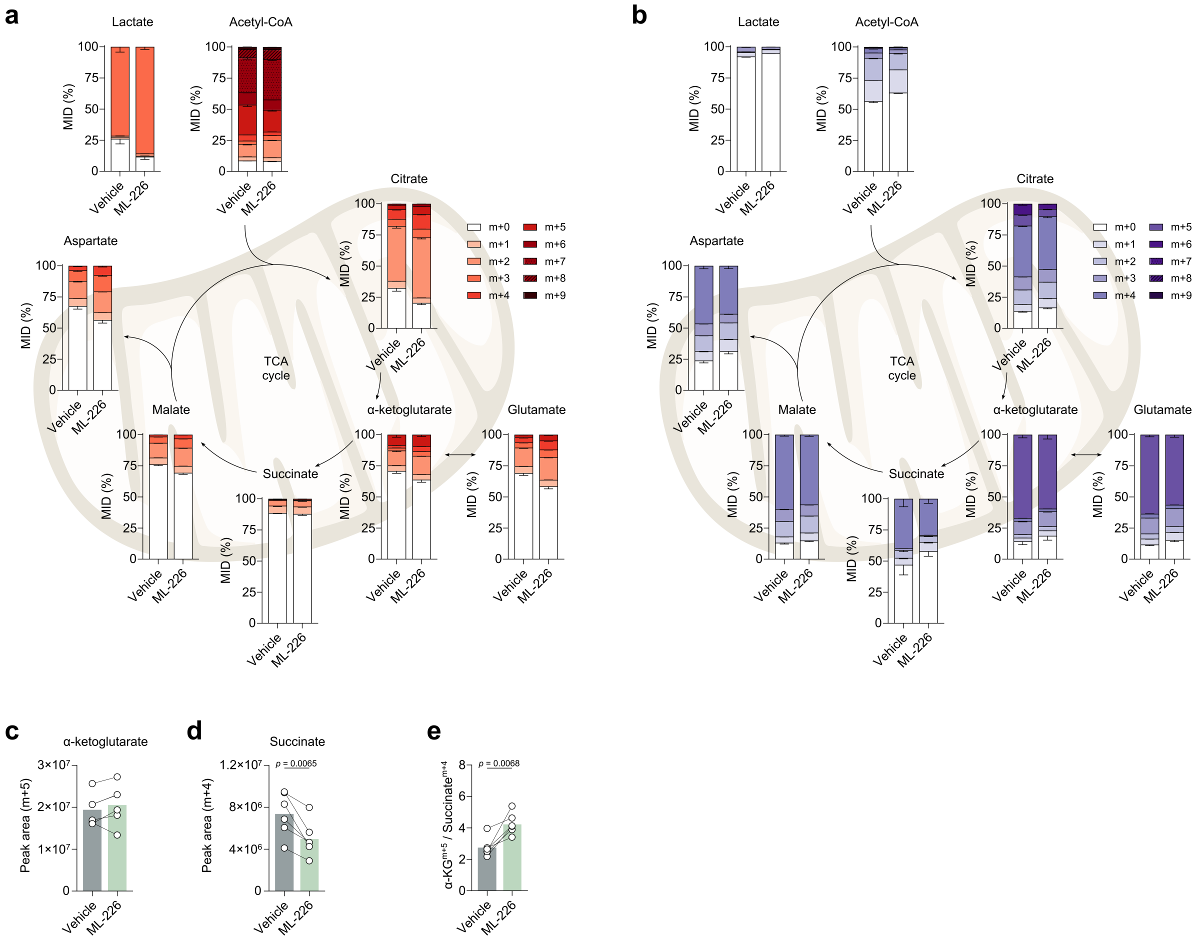

Supplementary Figure 3. ABHD11 inhibition alters glucose and glutamine utilisation (**a**,**b**) Stable isotope tracing of uniformly labelled (**a**) $^{13}C_6$-glucose or (**b**) $^{13}C_5$-glutamine into the TCA cycle and related intermediates in CD4+ effector T-cells (n = 6). Metabolites include: lactate, acetyl-CoA, citrate, α-ketoglutarate, glutamate, succinate, malate and aspartate. Mass isotopologue distribution (MID) represented as the proportion of the metabolite pool. (**c**) Intracellular levels of m+5 α-ketoglutarate (α-KG) in CD4+ T effector cells (n = 6). (**d**) Intracellular levels of m+4 succinate in CD4+ T effector cells (n = 6). (**e**) Determination of intracellular m+5 α-ketoglutarate to m+4 succinate ratio in CD4+ effector T-cells (n = 6). All experiments were carried out using human samples. CD4+ T-cells were activated with α-CD3 (2 µg/ml) and α-CD28 (20 µg/ml) for 24 h, in the presence and absence of ML-226. Statistical tests used: two-tailed paired T-test (**c**-**e**). Data are expressed as either: mean ± SEM; or mean, with paired dots representing biological replicates. Source data are provided as a Source Data file.

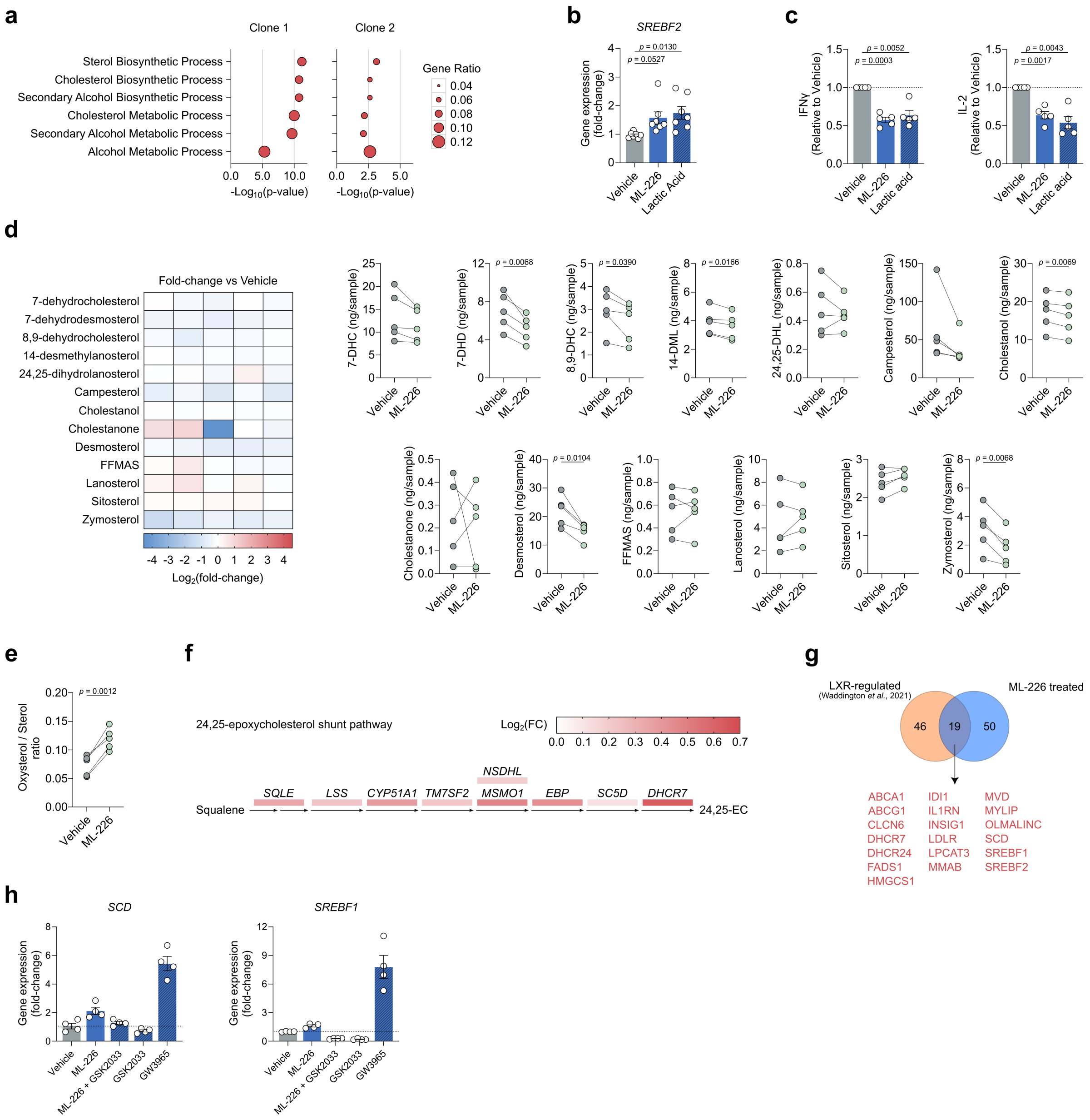

Supplementary Figure 4. ABHD11 inhibition reduces non-oxygenated sterol levels and drives activation of a mevalonate shunt pathway

(Figure legend on next page)

Supplementary Figure 4. ABHD11 inhibition reduces non-oxygenated sterol levels and drives activation of a mevalonate shunt pathway

(**a**) Pathway enrichment analysis based on differentially-expressed genes in ABHD11 knockdown Jurkat T-cells (n = 3). Selected metabolism-associated pathways amongst the top 10 enriched pathways are shown. (**b**) qPCR analysis of *SREBF2* expression in CD4+ T-cells, activated in the presence and absence of ML-226 or lactic acid (n = 7). (**c**) IL-2 and IFNγ production by CD4+ T-cells, activated in the presence and absence of ML-226 or lactic acid (n = 5). (**d**) Intracellular levels of selected non-oxygenated sterols in CD4+ T-cells. Metabolites include: 7-dehydrocholesterol (7-DHC), 7-dehydrodesmosterol (7-DHD), 8,9-dehydrocholesterol (8,9-DHC), 14-desmethylanosterol (14-DML), 24,25-dihydrolanosterol (24,25-DHL), campesterol, cholestanol, cholestanone, desmosterol, follicular fluid meiosis-activating sterol (FFMAS), lanosterol, sitosterol, zymosterol (n = 5). Heatmap represented as Log_2(fold-change) versus vehicle control. (**e**) Oxysterol / sterol ratio in CD4+ T-cells (n = 5). (**f**) Changes in enzyme transcript levels within the 24,25-epoxycholesterol shunt pathway, as measured by RNA-seq, in CD4+ T-cells (n = 4). (**g**) Overlap between liver X receptor-associated genes and genes differentially-regulated by ABHD11 inhibition in CD4+ T-cells (n = 4). (**h**) qPCR analysis of *SCD* and *SREBF1* expression in CD4+ T-cells, activated in the presence and absence of ML-226, GSK2033 and GW3965 (n = 4). All experiments were carried out using human samples. CD4+ T-cells were activated with α-CD3 (2 μg/ml) and α-CD28 (20 μg/ml) for 24 h. Data are expressed as either: mean, with paired dots representing biological replicates; or mean ± SEM. Statistical tests used: one-way ANOVA with Dunnett's multiple comparisons test (**b**), two-tailed one-sample T-test (**c**), two-tailed paired T-test (**d**, **e**) Source data are provided as a Source Data file.

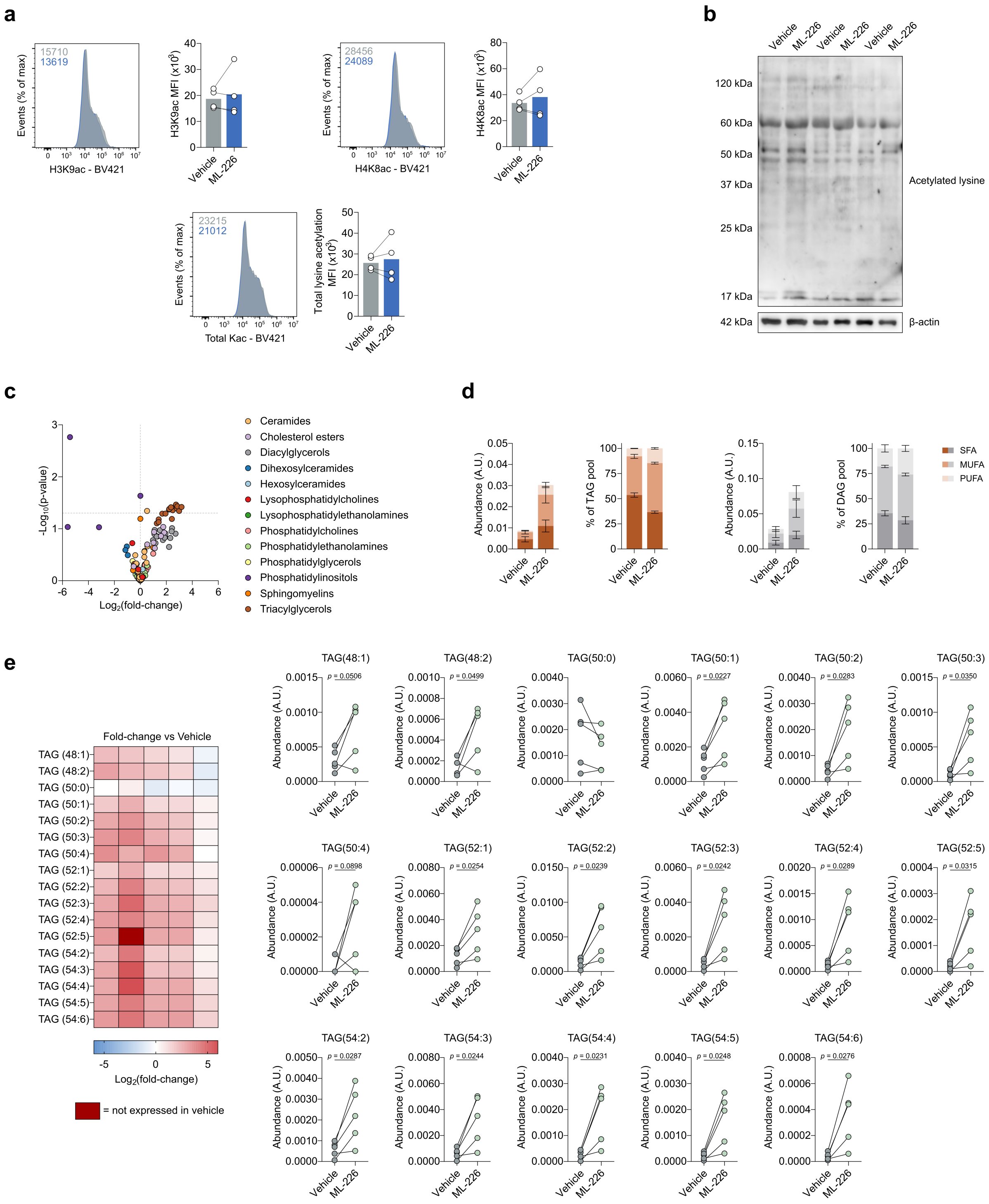

Supplementary Figure 5. ABHD11 inhibition increases triacylglycerol levels
(Figure legend on next page)

Supplementary Figure 5. ABHD11 inhibition increases triacylglycerol levels

(**a**) Intracellular histone acetylation levels, as measured by flow cytometry, in CD4+ effector T-cells. Histone acetylation measured on H3K9 and H4K8. Total lysine acetylation measured as a control (n = 4). (**b**) Total lysine acetylation in CD4+ T-cells (n = 3). Protein loading assessed using β-actin. (**c**) Differential lipid analysis by LC-MS/MS in CD4+ effector T-cells (n = 5). (**d**) Total and relative abundance of triacylglycerols (TAGs; brown) and diacylglycerols (DAGs; grey) in CD4+ T-cells (n = 5). (**e**) Intracellular levels of TAGs in CD4+ T-cells. Metabolites include: TAG(48:1), TAG(48:2), TAG(50:0), TAG(50:1), TAG(50:2), TAG(50:3), TAG(50:4), TAG(52:1), TAG(52:2), TAG(52:3), TAG(52:4), TAG(52:5), TAG(54:2), TAG(54:3), TAG(54:4), TAG(54:5), TAG(54:6). Heatmap represented as Log_2(fold-change) versus vehicle control (n = 5). All experiments were carried out using human samples. CD4+ T-cells were activated with α-CD3 (2 μg/ml) and α-CD28 (20 μg/ml) for 24 h, in the presence and absence of ML-226. Data are expressed as mean, with paired dots representing biological replicates. Statistical tests used: two-tailed paired T-test (**a**, **e**). Source data are provided as a Source Data file.

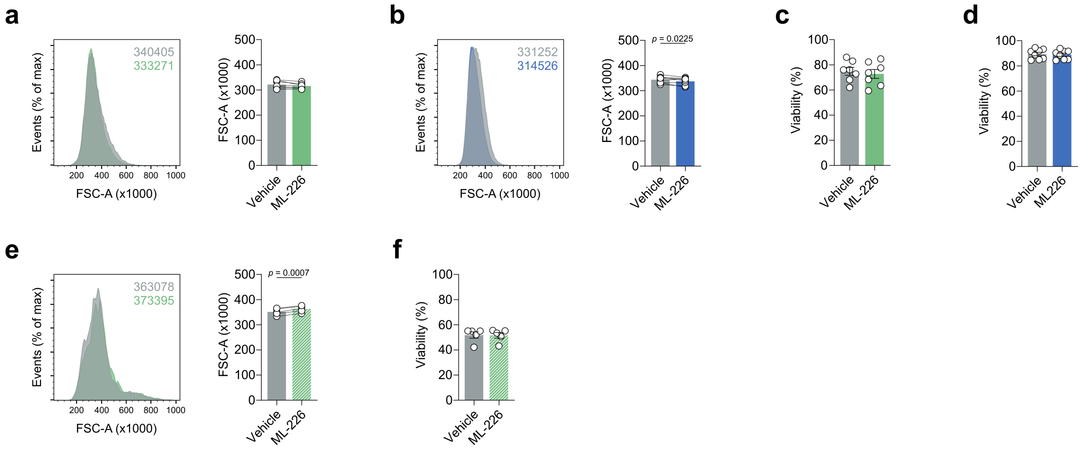

Supplementary Figure 6. ABHD11 inhibition has no clear effect on T-cell size and viability in autoimmune patient cohorts

(**a**,**b**) Cell size, as determined by forward scatter area, of patient-derived CD4+ T-cells from (**a**) rheumatoid arthritis (RA; n = 7) and (**b**) type 1 diabetes (T1D; n = 8) cohorts. (**c**,**d**) Cell viability, as determined by DRAQ7®, in patient-derived CD4+ T-cells in (**c**) RA (n = 7) and (**d**) T1D (n = 8) cohorts. (**e**) Cell size, as determined by forward scatter area, of patient-derived synovial fluid mononuclear cells (SFMCs; n = 5). (**f**) Cell viability, as determined by DRAQ7®, in patient-derived SFMCs (n = 5). All experiments were carried out using human samples. CD4+ T-cells were activated with α-CD3 (2 µg/ml) and α-CD28 (20 µg/ml) for 24 h, in the presence and absence of ML-226, unless otherwise stated. Data are expressed as either: mean, with paired dots representing biological replicates; or mean ± SEM. Statistical tests used: two-tailed paired T-test (**a**, **b**, **e**), two-tailed unpaired T-test (**c**, **d**, **f**). Source data are provided as a Source Data file.

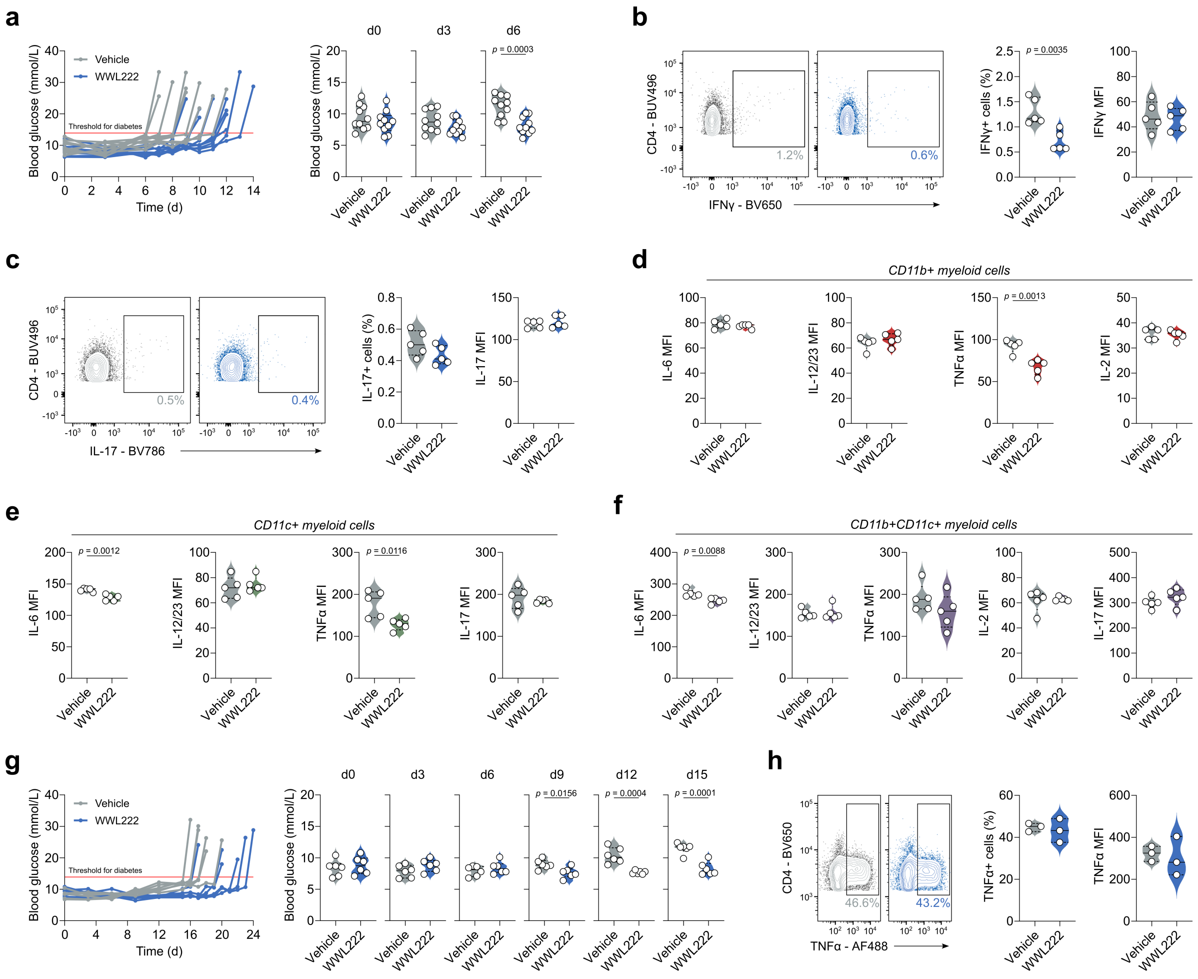

Supplementary Figure 7. ABHD11 inhibition delays T1D by stabilising blood glucose levels and altering the cytokine profile

(**a**) Blood glucose values in the presence and absence of daily injections i.p of 2.5 mg/kg WWL222 during an *in vivo* diabetes adoptive transfer model using BDC2.5 HIP-activated BDC2.5 CD4+ T cells (n = 10). For blood glucose traces, each line represents a distinct biological replicate, with dots representing blood glucose measurements taken. Red line indicates the threshold for diabetes. (**b**,**c**) Intracellular (**b**) IFNγ and (**c**) IL-17 expression by CD4+ T-cells (n = 5). (**d**) Intracellular IL-6, IL-12/23, TNFα and IL-2 expression by splenic $CD11b^{+}$ myeloid cells (n = 5). (**e**) Intracellular IL-6, IL-12/23, TNFα and IL-17 expression by splenic $CD11c^{+}$ myeloid cells (n = 5). (**f**) Intracellular IL-6, IL-12/23, TNFα, IL-2 and IL-17 expression by splenic $CD11b^{+}CD11c^{+}$ myeloid cells (n = 5). (**g**) Blood glucose values in the presence and absence of daily injections i.p of 2.5 mg/kg WWL222 during an *in vivo* diabetes adoptive transfer model using splenocytes (n = 6). For blood glucose traces, each line represents a distinct biological replicate, with dots representing blood glucose measurements taken. Red line indicates the threshold for diabetes. (**h**) Intracellular TNFα expression by CD4+ T-cells (n = 5). All experiments were carried out using murine samples. Mice were injected daily with the indicated dose of WWL222. Data are expressed as median ± interquartile range. Statistical tests used: two-tailed unpaired T-test (**a**-**h**). Source data are provided as a Source Data file.

a

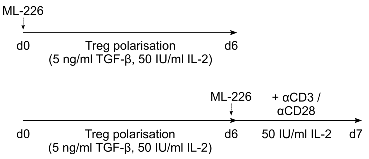

Supplementary Figure 8. Regulatory T cell polarisation

(**a**) Schematic overview of regulatory T cell (Treg) polarisation experiments in CD4+ naïve T cells.

a

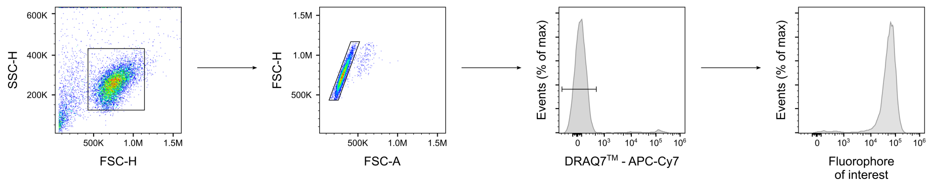

Supplementary Figure 9. Representative gating strategy

(**a**) Representative gating strategy employed for flow cytometry analysis. Cell doublets were excluded from analysis based on forward scatter-height versus forward scatter-area. Cell death was monitored using DRAQ7™ (1 µM; Biostatus, UK) and dead cells were excluded from analysis.

Supplementary Table 1. Rheumatoid arthritis patient demographics

Patient demographics	Rheumatoid arthritis<br>PBMCs<br>(n = 7)	 SFMCs<br>(n = 5)
**Age**		
Mean (SD)	68.6 (± 6.45)	66.0 (± 5.05)
Median	68.0	66.0
Range	57.0 – 77.0	60.0 – 71.0
**Sex** n (%)		
Female	5 (71.4%)	3 (60.0%)
Male	2 (28.6%)	2 (40.0%)
Not determined	0 (0.0%)	0 (0.0%)
**Treatment**		
No medication	1 (14.2%)	2 (40.0%)
bDMARD only	4 (57.1%)	1 (20.0%)
csDMARD only	2 (28.6%)	1 (20.0%)
bDMARD + csDMARD	0 (0.0%)	0 (0.0%)
bDMARD + TNF inhibitor	0 (0.0%)	0 (0.0%)
Not determined	0 (0.0%)	1 (20.0%)
**Rheumatoid Factor**		
Positive	4 (57.1%)	0 (0.0%)
Negative	3 (42.9%)	0 (0.0%)
Not determined	0 (0.0%)	5 (100.0%)
**ACPA**		
Positive	4 (57.1%)	0 (0.0%)
Negative	3 (42.9%)	0 (0.0%)
Not determined	0 (0.0%)	5 (100.0%)
**DAS28**		
Mean (SD)	4.59 (± 2.57)	3.56 (± 0.30)
Median	3.84	3.64
Range	2.47 – 7.60	3.13 – 3.83

ACPA, anti-citrullinated protein antibodies; DAS28, disease activity score; DMARD, disease-modifying antirheumatic drugs; PBMCs, peripheral blood mononuclear cells; SFMCs, synovial fluid mononuclear cells

Supplementary Table 2. Type 1 diabetes patient demographics.

Patient demographics	Type 1 diabetes (n = 8)
**Age**	
Mean (SD)	36.4 (± 14.3)
Median	32.4
Range	19.0 – 62.0
**Sex** n (%)	
Female	7 (87.5%)
Male	1 (12.5%)
**Diabetes duration**	
Mean (SD)	14.2 (± 14.8)
Median	11.06
Range	1.8 – 47.0
**Treatment**	
Insulin	8 (100.0%)

Supplementary Table 3. CRISPR single guide RNA sequences

	Sequence
Sequence 1	AAGATCTTGGCCCAGCAGAC
Sequence 2	GCAGAAGGTCCTGCAGGTCC

Supplementary Table 4. Primer sequences

Primer	Sequence
IFNG (F)	TCAGCTCTGCATCGTTTTGG
IFNG (R)	TGGTCTCCACACTCTTTTGG
IL2 (F)	CCTCAACTCCTGCCACAATG
IL2 (R)	TGTGAGCATCCTGGTGAGTT
IL10 (F)	CCTGCCTAACATGCTTCGAG
IL10 (R)	AAGAAATCGATGACAGCGCC
IL17 (F)	GCACAAACTCATCCATCCCC
IL17 (R)	TCCTCATTGCGGTGGAGATT
RPL19 (F)	GCGAGCTCTTTCCTTTCGCT
RPL19 (R)	TGCTGACGGGAGTTGGCATT
SCD (F)	AGACGATGCCCCTCTACTTG
SCD (R)	CTCCACAGACGATGAGCTCC
SREBF1 (F)	GAGCCACCCTTCAGCGAG
SREBF1 (R)	AAGGCTTCAAGAGAGGAGCT
SREBF2 (F)	TGGAGACCATGGAGACCCT
SREBF2 (R)	TGCTACCACTACCACCACTG

Uncropped blots

Supplementary Figure 1D

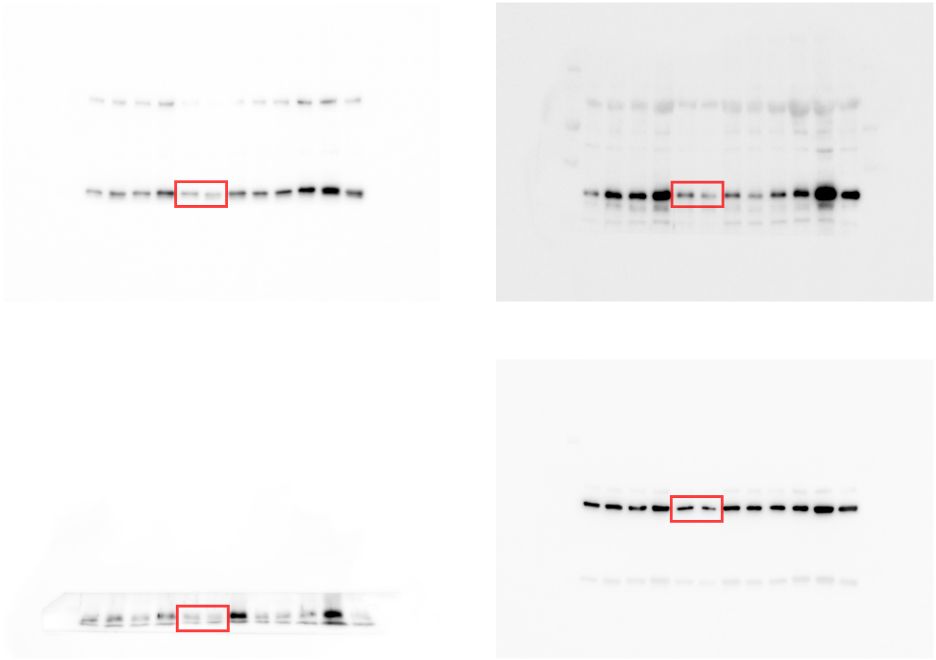

Supplementary Figure 5B

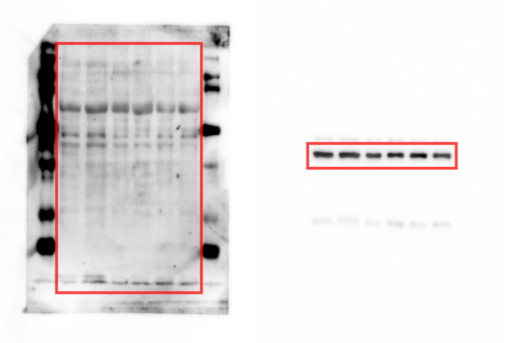

Supplement: Supplementary file 1 — Supplementary Information [file 41467_2025_65417_MOESM1_ESM.pdf]
