# Supplementary material for: Mitochondrial ABHD11 inhibition drives sterol metabolism to modulate T-cell effector function
Source: Nat Commun. 2025 Nov 3;16:9484. doi: 10.1038/s41467-025-65417-4 (PMC12583646; doi:10.1038/s41467-025-65417-4)
Supplement: Supplementary file 2 — Reporting Summary [file 41467_2025_65417_MOESM2_ESM.pdf]

nature portfolio

Corresponding author(s): James Pearson, Emma Vincent, Nicholas Jones

Last updated by author(s): 02/09/2025

Reporting Summary

Nature Portfolio wishes to improve the reproducibility of the work that we publish. This form provides structure for consistency and transparency in reporting. For further information on Nature Portfolio policies, see our Editorial Policies and the Editorial Policy Checklist.

Statistics

For all statistical analyses, confirm that the following items are present in the figure legend, table legend, main text, or Methods section.

n/a	Confirmed	
☐	☒	The exact sample size (*n*) for each experimental group/condition, given as a discrete number and unit of measurement
☐	☒	A statement on whether measurements were taken from distinct samples or whether the same sample was measured repeatedly
☐	☒	The statistical test(s) used AND whether they are one- or two-sided<br>*Only common tests should be described solely by name; describe more complex techniques in the Methods section.*
☒	☐	A description of all covariates tested
☐	☒	A description of any assumptions or corrections, such as tests of normality and adjustment for multiple comparisons
☐	☒	A full description of the statistical parameters including central tendency (e.g. means) or other basic estimates (e.g. regression coefficient) AND variation (e.g. standard deviation) or associated estimates of uncertainty (e.g. confidence intervals)
☐	☒	For null hypothesis testing, the test statistic (e.g. *F*, *t*, *r*) with confidence intervals, effect sizes, degrees of freedom and *P* value noted<br>*Give P values as exact values whenever suitable.*
☒	☐	For Bayesian analysis, information on the choice of priors and Markov chain Monte Carlo settings
☒	☐	For hierarchical and complex designs, identification of the appropriate level for tests and full reporting of outcomes
☒	☐	Estimates of effect sizes (e.g. Cohen's *d*, Pearson's *r*), indicating how they were calculated

Our web collection on statistics for biologists contains articles on many of the points above.

Software and code

Policy information about availability of computer code

Data collection

Flow cytometry data were acquired on the Novocyte 3000 (Agilent), FACS Canto II or Symphony A3 Cell Analyser (both BD). Metabolic data were acquired using a Seahorse XFe extracellular flux analyser (Agilent).
Metabolite profiling and identification was achieved using a Q Exactive Plus Orbitrap mass spectrometer (ThermoFisher). Lipids and sterols were analysed using a SCIEX QTRAP 6500+ (SCIEX).
RNA-seq libraries were sequenced on Illumina NovaSeq 6000 as paired-end 150-nt reads.

Data analysis

Statistical analysis was performed using GraphPad Prism version 10 (USA).

Flow cytometry data analysis was performed using FlowJo version 10 (TreeStar).

LC-MS data analysis was undertaken in Skyline (version 23.1.0.455).

LS-MS/MS data was analysed using MultiQuant software (SCIEX).

RNA-seq analysis:
For human, RNA-Seq analysis, Fastq files were quality assessed and trimmed using FastP(v0.23.1), before reads were mapped to the genome GRCh38 using STAR (Spliced Transcripts Alignment to a Reference; v2.7.9a) with 2-pass method and multimapping set to 1. Featurecounts(v2.0.3) was used to generate count files for each sample, with counting performed at the gene level. Differential gene expression analysis was calculated via eBayesian fit of TMM (Trimmed Mean of M-values) using an EdgeR workflow of Limma-Voom(v3.58.1). Genes were filtered for an adjusted p-value < 0.05 and over representation analysis was performed using gProfiler (v.e111.eg58.p18.f463989d) for Gene Ontology terms for Biological Processes (GO:BP). ReViGo(v1.8.1) was used to reduce the repetition of

canonical pathway terms. GeneSet enrichment analysis was performed using genekitR (v1.2.5). Protein-protein interaction was assessed using StringDB(v12.0), whereby differentially expressed genes were treated as if they were fully transcribed into proteins. Transcription factor enrichment analysis was performed using X2Kweb (v14/22 Appyter), which infers upstream regulatory networks from the differentially-expressed gene signature.

For RNA-Seq analysis on ABHD11 knockdown clones, sequencing data were processed using the nf-core/nanoseq pipeline (v3.1.0) with the cDNA protocol. Demultiplexing was skipped, and reads were aligned to the human reference genome (Gencode Release 45, GRCh38.p14) using minimap2. Transcript quantification was performed with bambu. Differential expression analysis was conducted in R (v4.3.2) using DESeq2 (v1.42.1). Data visualisations and exploratory analyses were performed with tidyverse (v2.0.0) and clusterProfiler (v4.10.1).

For manuscripts utilizing custom algorithms or software that are central to the research but not yet described in published literature, software must be made available to editors and reviewers. We strongly encourage code deposition in a community repository (e.g. GitHub). See the Nature Portfolio guidelines for submitting code & software for further information.

Data

Policy information about availability of data

All manuscripts must include a data availability statement. This statement should provide the following information, where applicable:

- Accession codes, unique identifiers, or web links for publicly available datasets
- A description of any restrictions on data availability
- For clinical datasets or third party data, please ensure that the statement adheres to our policy

All data are available upon request and can be found within the manuscript and supplementary information. The raw RNA-Seq data generated in this study have been deposited in the European Genome-phenome Archive (EGA) under the accession code EGAS50000001297. Mass spectrometry data are available through https://massive.ucsd.edu/ (dataset: MSV000099454) Source data are provided with this paper.

Research involving human participants, their data, or biological material

Policy information about studies with human participants or human data. See also policy information about sex, gender (identity/presentation), and sexual orientation and race, ethnicity and racism.

Reporting on sex and gender	We have reported the sex of autoimmune patient cohorts (type 1 diabetes and rheumatoid arthritis in the supplementary (tables 1 and 2).
Reporting on race, ethnicity, or other socially relevant groupings	The participant sample was drawn from Swansea University's student and staff population, within which non-white ethnicities account for around 30%. In terms of ethnicity, this population is more diverse than that of the UK as a whole, within which non-white ethnicities account for less than 20% of people (Census 2021). We did not collect race or ethnicity from type 1 diabetes or rheumatoid arthritis individuals.
Population characteristics	The study was performed on a population of healthy adults aged 18-70 years old and included men and women. Participants were excluded if they had an immune-mediated disease, cancer in the past 5 years or had current/recent symptoms of viral or other infection. Participants using medication, such as statins, with immune response modifying effects, were also excluded. All samples were collected between 0800 and 1200. Patient characteristics are found in the Supplementary.
Recruitment	Healthy participants were recruited from the staff and student populations at Swansea University, Wales UK. Potential participants responded to ethics committee approved advertising by contacting the local clinical research facility. The clinical research facility oversaw recruitment through informed written consent in response to an ethically approved participant information sheet that explained the study. Participant recruitment was conducted by the Joint Clinical Research Facility at Swansea University with no selection bias. Autoimmune patients were recruited in clinics.
Ethics oversight	For healthy controls, informed written consent and ethical approval was obtained from Swansea University Medical School Research Ethics Committee (SUMSRESC; 2022-0029).<br><br>For autoimmune patient cohorts:<br>Rheumatoid arthritis (ethics RS18-055)<br>Type 1 diabetes mellitus (ethics 12/WA/0033)

Note that full information on the approval of the study protocol must also be provided in the manuscript.

Field-specific reporting

Please select the one below that is the best fit for your research. If you are not sure, read the appropriate sections before making your selection.

☒ Life sciences ☐ Behavioural & social sciences ☐ Ecological, evolutionary & environmental sciences

For a reference copy of the document with all sections, see nature.com/documents/nr-reporting-summary-flat.pdf

Life sciences study design

All studies must disclose on these points even when the disclosure is negative.

Sample size	Nonstatistical test was used to determine sample size. For standard experimental procedures, sample sizes from our previous experience

Sample size	were used (Jones et al., 2019, Nat Comms, Jenkins et al., 2023 Cell Metabolism). Experiments were performed using sample sizes based on standard protocols in the field (see Diehl et al. Nat Metab. 2019 Sep;1(9):861-867, Luengo et al. Nat Commun. 2019 Dec 6;10(1):5604). In vivo transfer experiments were performed using sample sizes based on previous experimental designs (Pearson et al., Front Immunol., 2023; Huang et al, Front Immunol., 2021; Huang et al, Cellular & Molecular Immunology, 2021; Sha et al., Diabetes, 2020)
Data exclusions	No data was excluded
Replication	All experiments were replicated as described in the figure legend
Randomization	Female mice from different breeders were randomised for study either as donors or recipients.
Blinding	Blinding: Post-randomisation, mice were blinded to the investigators and only unblinded at the end of the study after all analysis was completed.

Behavioural & social sciences study design

All studies must disclose on these points even when the disclosure is negative.

Study description	*Briefly describe the study type including whether data are quantitative, qualitative, or mixed-methods (e.g. qualitative cross-sectional, quantitative experimental, mixed-methods case study).*
Research sample	*State the research sample (e.g. Harvard university undergraduates, villagers in rural India) and provide relevant demographic information (e.g. age, sex) and indicate whether the sample is representative. Provide a rationale for the study sample chosen. For studies involving existing datasets, please describe the dataset and source.*
Sampling strategy	*Describe the sampling procedure (e.g. random, snowball, stratified, convenience). Describe the statistical methods that were used to predetermine sample size OR if no sample-size calculation was performed, describe how sample sizes were chosen and provide a rationale for why these sample sizes are sufficient. For qualitative data, please indicate whether data saturation was considered, and what criteria were used to decide that no further sampling was needed.*
Data collection	*Provide details about the data collection procedure, including the instruments or devices used to record the data (e.g. pen and paper, computer, eye tracker, video or audio equipment) whether anyone was present besides the participant(s) and the researcher, and whether the researcher was blind to experimental condition and/or the study hypothesis during data collection.*
Timing	*Indicate the start and stop dates of data collection. If there is a gap between collection periods, state the dates for each sample cohort.*
Data exclusions	*If no data were excluded from the analyses, state so OR if data were excluded, provide the exact number of exclusions and the rationale behind them, indicating whether exclusion criteria were pre-established.*
Non-participation	*State how many participants dropped out/declined participation and the reason(s) given OR provide response rate OR state that no participants dropped out/declined participation.*
Randomization	*If participants were not allocated into experimental groups, state so OR describe how participants were allocated to groups, and if allocation was not random, describe how covariates were controlled.*

Ecological, evolutionary & environmental sciences study design

All studies must disclose on these points even when the disclosure is negative.

Study description	*Briefly describe the study. For quantitative data include treatment factors and interactions, design structure (e.g. factorial, nested, hierarchical), nature and number of experimental units and replicates.*
Research sample	*Describe the research sample (e.g. a group of tagged Passer domesticus, all Stenocereus thurberi within Organ Pipe Cactus National Monument), and provide a rationale for the sample choice. When relevant, describe the organism taxa, source, sex, age range and any manipulations. State what population the sample is meant to represent when applicable. For studies involving existing datasets, describe the data and its source.*
Sampling strategy	*Note the sampling procedure. Describe the statistical methods that were used to predetermine sample size OR if no sample-size calculation was performed, describe how sample sizes were chosen and provide a rationale for why these sample sizes are sufficient.*
Data collection	*Describe the data collection procedure, including who recorded the data and how.*
Timing and spatial scale	*Indicate the start and stop dates of data collection, noting the frequency and periodicity of sampling and providing a rationale for these choices. If there is a gap between collection periods, state the dates for each sample cohort. Specify the spatial scale from which the data are taken*
Data exclusions	*If no data were excluded from the analyses, state so OR if data were excluded, describe the exclusions and the rationale behind them, indicating whether exclusion criteria were pre-established.*

Reproducibility	*Describe the measures taken to verify the reproducibility of experimental findings. For each experiment, note whether any attempts to repeat the experiment failed OR state that all attempts to repeat the experiment were successful.*
Randomization	*Describe how samples/organisms/participants were allocated into groups. If allocation was not random, describe how covariates were controlled. If this is not relevant to your study, explain why.*
Blinding	*Describe the extent of blinding used during data acquisition and analysis. If blinding was not possible, describe why OR explain why blinding was not relevant to your study.*

Did the study involve field work? ☐ Yes ☐ No

Field work, collection and transport

Field conditions	*Describe the study conditions for field work, providing relevant parameters (e.g. temperature, rainfall).*
Location	*State the location of the sampling or experiment, providing relevant parameters (e.g. latitude and longitude, elevation, water depth).*
Access & import/export	*Describe the efforts you have made to access habitats and to collect and import/export your samples in a responsible manner and in compliance with local, national and international laws, noting any permits that were obtained (give the name of the issuing authority, the date of issue, and any identifying information).*
Disturbance	*Describe any disturbance caused by the study and how it was minimized.*

Reporting for specific materials, systems and methods

We require information from authors about some types of materials, experimental systems and methods used in many studies. Here, indicate whether each material, system or method listed is relevant to your study. If you are not sure if a list item applies to your research, read the appropriate section before selecting a response.

Materials & experimental systems

n/a	Involved in the study	
☐	☒	Antibodies
☐	☒	Eukaryotic cell lines
☒	☐	Palaeontology and archaeology
☐	☒	Animals and other organisms
☒	☐	Clinical data
☒	☐	Dual use research of concern
☒	☐	Plants

Methods

n/a	Involved in the study	
☒	☐	ChIP-seq
☐	☒	Flow cytometry
☒	☐	MRI-based neuroimaging

Antibodies

Antibodies used

T cell activation:
T cells were activated with plate-bound anti-CD3 (2 µg/ml; OKT3; BioLegend) and soluble anti-CD28 (20 µg/ml; CD28.2; BioLegend).

Surface staining:
For human T cells, antibodies were used as follows: anti-CD25 (Pacific Blue, mIgG1κ, BC96, 302627), anti-CD25 (PE-Vio 615, rhIgG1, REA570, 130-123-035; Miltenyi), anti-CD44 (FITC, rhIgG1, REA690, 130-113-341; Miltenyi), anti-CD44 (Pacific Blue, mIgG1κ, BJ18, 338823), anti-CD69 (APC, mIgG1κ, FN50, 310910), anti-CD69 (FITC, mIgG1κ, FN50, 310904). Antibodies were purchased from BioLegend, unless otherwise stated. Human CD4+ T cell purity was monitored using anti-CD3 (Brilliant Violet 570™, mIgG1κ, UCHT1, 300436, Biolegend) and anti-CD4 (AlexaFluor® 647, mIgG2b, OKT4, 317422, BioLegend). CD4+ effector T cell purity was monitored using anti-CD4 (AlexaFluor® 647, mIgG2b, OKT4, 317422, BioLegend), anti-CD45RA (Brilliant Violet 605™, mIgG2b, HI100, 304134, BioLegend), anti-CD45RO (FITC, mIgG2a, UCHL1, 304204, BioLegend) and anti-CD197 (Pacific Blue, mIgG2a, G043H7, 353210, BioLegend).

For murine splenic T cells from BDC2.5 TCR transgenic NOD mice, antibodies were used as follows: anti-CD4 (BUV496, LewIgG2b, GK1.5, 612952; BD), anti-CD8 (PerCP/Cy5.5, rIgG2aκ, 53-6.7, 100734), anti-CD11b (Brilliant Violet 510™, rIgG2bκ, M1/70, 101263), anti-CD11c (BUV661, ahIgG2, N418, 750449; BD), anti-CD19 (APC-Cy7, rIgG2aκ, 1D3, 152412), anti-CD25 (APC, rIgG1, PC61, 102012), anti-CD40 (BUV615, LouIgG2aκ, 3/23, 751646; BD), anti-CD44 (BUV805, rIgG2bκ, IM7, 741921; BD), anti-CD62L (BUV395, rIgG2aκ, MEL-14, 569400; BD), anti-CD69 (BUV563, ahIgG1λ3, H1.2F3, 612952; BD), anti-CD80 (AlexaFluor® 594, rrIgG, 2740B, FAB7401T-100ug; Bio-Techne), anti-CD86 (BUV737, rIgG2bκ, PO3, 741757; BD), anti-MHC-I-[H-2kd] (Brilliant Violet 421™, mIgG2aκ, SF1-1.1, 116623) and anti-MHC-II-[I-AK] (PE, mIgG2aκ, 10-3.6, 109908). Antibodies were purchased from BioLegend, unless otherwise stated.

Intracellular staining:

For human T cells, primary antibodies were used as follows: anti-Histone H3 (acetyl K9; H3K9ac, ab12178), anti-Histone H4 (acetyl K8; H4K8ac, ab15823) and anti-acetyl lysine (ab21623). Primary antibodies were purchased from Abcam. Secondary antibodies were used as follows: anti-rabbit IgG secondary antibody (Brilliant Violet 421™, donkey pIg, Poly4064, 406410; BioLegend).

For murine T cells from C57BL/6 mice spleens, antibodies were used as follows: anti-CD4 (Brilliant Violet 758™, rIgG2aκ, RM4-5, 100551; BioLegend), anti-IL-13 (FITC, rIgG1κ, eBio13A, 53-7133-82; Invitrogen), anti-IL-17A (PE, rIgG1κ, TC11-18H10, 561020; BD Pharmigen), anti-IFNγ (eFluor™ 450, rIgG1κ, XMG1.2, 48-7311-82; Invitrogen) and anti-FOXP3 (PE, rIgG2ακ, FJK-16S, 12-5773-82; eBioscience).

For murine T cells from BDC2.5 TCR transgenic NOD mice spleens, antibodies were used as follows: anti-IL-2 (Spark Red™ 718, rIgG2bκ, JES6-5H4, 503852), anti-IL-6 (APC, rIgG1κ, MP5-20F3, 503852), anti-IL-10 (Brilliant Violet 605™, rIgG2bκ, JES5-16E3, 505031), anti-IL-12/23 (PE-Cy7, rIgG2κ, C15.6, 505210), anti-IL-17a (Brilliant Violet 786™, rIgG2κ, TC11-18H10.1, 506928), anti-IFNγ (Brilliant Violet 650™, rIgG1κ, XMG1.2, 505832) and anti-TNFα (Brilliant Violet 750™, rIgG1κ, MP6-XT22, 506358). Antibodies were purchased from BioLegend, unless otherwise stated.

For murine splenocytes from NOD mice spleens, antibodies were used as follows: anti-IFNγ (Brilliant Violet 605™, rIgG1κ, XMG1.2, 505840), anti-TNFα (Alexa Fluor® 488, rIgG1κ, MP6-XT22, 506313).

Immunoblotting:
For immunoblotting, antibodies were used as follows: ABHD11 (PA5-54962; Invitrogen), acetylated lysine (MA5-33031; Invitrogen), β-actin (ab8226; Abcam), Bcl-2 (1507), Caspase-3 (9662), cleaved Caspase-3 (9661), phospo-LATTyr220 (3584), LAT (45533), phospho-PLCγTyr783 (14008), PLCγ (5690), phospho-ZAP70Tyr493/Tyr526 (2704), ZAP70 (3165). All antibodies were purchased from Cell Signaling, unless otherwise stated.

Validation

All antibodies are commercially available. Antibodies employed here in our manuscript were previously reported and routinely used for the application used. All companies used report quality control measures to ensure validity and reproducibility. Validation information and previous citations for each individual antibody are found in the data sheets provided by the company.

Eukaryotic cell lines

Policy information about cell lines and Sex and Gender in Research

Cell line source(s)	Jurkat E6.1 T-cells
Authentication	None of the cell lines used were authenticated.
Mycoplasma contamination	All cell lines tested negative for mycoplasma contamination.
Commonly misidentified lines (See ICLAC register)	*Name any commonly misidentified cell lines used in the study and provide a rationale for their use.*

Palaeontology and Archaeology

Specimen provenance	*Provide provenance information for specimens and describe permits that were obtained for the work (including the name of the issuing authority, the date of issue, and any identifying information). Permits should encompass collection and, where applicable, export.*
Specimen deposition	*Indicate where the specimens have been deposited to permit free access by other researchers.*
Dating methods	*If new dates are provided, describe how they were obtained (e.g. collection, storage, sample pretreatment and measurement), where they were obtained (i.e. lab name), the calibration program and the protocol for quality assurance OR state that no new dates are provided.*

☐ Tick this box to confirm that the raw and calibrated dates are available in the paper or in Supplementary Information.

Ethics oversight	*Identify the organization(s) that approved or provided guidance on the study protocol, OR state that no ethical approval or guidance was required and explain why not.*

Note that full information on the approval of the study protocol must also be provided in the manuscript.

Animals and other research organisms

Policy information about studies involving animals; ARRIVE guidelines recommended for reporting animal research, and Sex and Gender in Research

Laboratory animals

BDC2.5 TCR transgenic NOD mice (NOD.Cg-Tg(TcraBDC2.5,TcrbBDC2.5)1Doi/DoiJ; 004460)
Rag1-/-NOD mice (NOD.129S7(B6)-Rag1tm1Mom/J; 003729)
NOD mice originally from the NOD/Caj colony (MGI:3757672) have been maintained at Cardiff over 15 years.
All mice received water and irradiated food (T.2919.CS; ENVIGO) ad libitum and were housed at Cardiff University in specific-pathogen-free Scantainers with 12 h light–dark cycles.

Wild animals	There were no wild animals used in this study.
Reporting on sex	Female donors and recipients were used for these experiments.
Field-collected samples	No field-collected samples were used in this study.
Ethics oversight	All animal experiments were approved by the Cardiff University ethical review process and conducted under UK Home Office licence in accordance with the UK Animals (Scientific Procedures) Act 1986 and associated guidelines.

Note that full information on the approval of the study protocol must also be provided in the manuscript.

Clinical data

Policy information about clinical studies

All manuscripts should comply with the ICMJE guidelines for publication of clinical research and a completed CONSORT checklist must be included with all submissions.

Clinical trial registration	*Provide the trial registration number from ClinicalTrials.gov or an equivalent agency.*
Study protocol	*Note where the full trial protocol can be accessed OR if not available, explain why.*
Data collection	*Describe the settings and locales of data collection, noting the time periods of recruitment and data collection.*
Outcomes	*Describe how you pre-defined primary and secondary outcome measures and how you assessed these measures.*

Dual use research of concern

Policy information about dual use research of concern

Hazards

Could the accidental, deliberate or reckless misuse of agents or technologies generated in the work, or the application of information presented in the manuscript, pose a threat to:

No	Yes	
☐	☐	Public health
☐	☐	National security
☐	☐	Crops and/or livestock
☐	☐	Ecosystems
☐	☐	Any other significant area

Experiments of concern

Does the work involve any of these experiments of concern:

No	Yes	
☐	☐	Demonstrate how to render a vaccine ineffective
☐	☐	Confer resistance to therapeutically useful antibiotics or antiviral agents
☐	☐	Enhance the virulence of a pathogen or render a nonpathogen virulent
☐	☐	Increase transmissibility of a pathogen
☐	☐	Alter the host range of a pathogen
☐	☐	Enable evasion of diagnostic/detection modalities
☐	☐	Enable the weaponization of a biological agent or toxin
☐	☐	Any other potentially harmful combination of experiments and agents

Plants

Seed stocks: N/A

Novel plant genotypes: N/A

Authentication: N/A

ChIP-seq

Data deposition

☐ Confirm that both raw and final processed data have been deposited in a public database such as GEO.

☐ Confirm that you have deposited or provided access to graph files (e.g. BED files) for the called peaks.

Data access links
May remain private before publication.
For "Initial submission" or "Revised version" documents, provide reviewer access links. For your "Final submission" document, provide a link to the deposited data.

Files in database submission: *Provide a list of all files available in the database submission.*

Genome browser session (e.g. UCSC): *Provide a link to an anonymized genome browser session for "Initial submission" and "Revised version" documents only, to enable peer review. Write "no longer applicable" for "Final submission" documents.*

Methodology

Replicates: *Describe the experimental replicates, specifying number, type and replicate agreement.*

Sequencing depth: *Describe the sequencing depth for each experiment, providing the total number of reads, uniquely mapped reads, length of reads and whether they were paired- or single-end.*

Antibodies: *Describe the antibodies used for the ChIP-seq experiments; as applicable, provide supplier name, catalog number, clone name, and lot number.*

Peak calling parameters: *Specify the command line program and parameters used for read mapping and peak calling, including the ChIP, control and index files used.*

Data quality: *Describe the methods used to ensure data quality in full detail, including how many peaks are at FDR 5% and above 5-fold enrichment.*

Software: *Describe the software used to collect and analyze the ChIP-seq data. For custom code that has been deposited into a community repository, provide accession details.*

Flow Cytometry

Plots

Confirm that:

☒ The axis labels state the marker and fluorochrome used (e.g. CD4-FITC).

☒ The axis scales are clearly visible. Include numbers along axes only for bottom left plot of group (a 'group' is an analysis of identical markers).

☒ All plots are contour plots with outliers or pseudocolor plots.

☒ A numerical value for number of cells or percentage (with statistics) is provided.

Methodology

Sample preparation

Surface staining:
Flow cytometry was performed on T cells following cell culture. Cell death was monitored using DRAQ7™ (1µM, DR71000; Biostatus), unless otherwise stated, and dead cells were excluded from analysis. For human T cells, surface staining was performed at room temperature (RT) for 15 min in the dark.

For murine splenic T cells from C57BL/6 mice, cell death was monitored using the Zombie Aqua Fixable Viability Kit (BD).

For murine splenic T cells from BDC2.5 TCR transgenic NOD mice, single cell suspensions were incubated with TruStain FcX™ Fc Receptor Blocking Solution (clone 93; BioLegend) for 10 min at 4°C prior to staining for surface markers for 30 min at 4°C. Cell death was monitored using either the Zombie Aqua Fixable Viability Kit or Fixable Viability Stain 575V (both BD).

Intracellular staining:
For human T cells, intracellular markers were stained using the eBioscience™ Foxp3 / Transcription Factor Staining Buffer Set (00-5523-00) and cell death monitored using the eBioscience™ Fixable Viability Dye eFluor™ 506 (65-0866-14) as per the manufacturer's instructions (both Invitrogen). Following surface staining, cells were fixed for 30 min at RT before staining in permeablisation buffer. Primary antibodies were incubated for 1 h at RT, followed by incubation with an anti-rabbit IgG secondary antibody (Brilliant Violet 421™, donkey pIg, Poly4064, 406410; BioLegend) for 30 min at RT. Primary antibodies used were purchased from Abcam, unless otherwise stated: anti-Histone H3 (acetyl K9; H3K9ac, ab12178), anti-Histone H4 (acetyl K8; H4K8ac, ab15823) and anti-acetyl lysine (ab21623).

For murine T cells from C57BL/6 mice spleens, intracellular markers were stained using the Cytofix/Cytoperm Fixation/ Permeabilisation kit (BD) as per the manufacturer's instructions. 4 h prior to intracellular staining, cells were activated with PMA (50 ng/ml) and ionomycin (500 ng/ml), and protein transport blocked using monensin (3 µM; all Merck). Following viability staining, cells were fixed and permeabilised and stained.

For murine T cells from BDC2.5 TCR transgenic NOD mice spleens, intracellular markers were stained using the Cytofix/ Cytoperm Fixation/Permeabilisation kit (BD) as per the manufacturer's instructions. 3 h prior to intracellular staining, cells were activated with PMA (50 ng/ml) and ionomycin (500 ng/ml), and protein transport blocked using GolgiPlug (BD). Following surface staining, cells were fixed for 20 min at RT before permeabilisation. Cells were incubated with TruStain FcX™ as previously described prior to staining for intracellular cytokines.

For murine splenocytes from NOD mice spleens, intracellular markers were stained using the Cytofix/Cytoperm Fixation/ Permeabilisation kit (BD) as per the manufacturer's instructions. 3 h prior to intracellular staining, cells were activated with PMA (50 ng/ml) and ionomycin (500 ng/ml), and protein transport blocked using GolgiPlug (BD). Following surface staining, cells were fixed for 20 min at RT before permeabilisation. Cells were incubated with TruStain FcX™ as previously described prior to staining for intracellular cytokines.

Puromycin incorporation:
Protein translation was assessed using anti-puromycin (AlexaFluor® 488, 12D10, MABE343-AF488; Merck). Puromycin (10 µM; Merck) was added 15 min prior to the end of 4 h and 24 h T cell activation. Cells were washed in ice-cold PBS before intracellular staining was performed using Inside Stain Kit (Miltenyi) as per the manufacturer's instructions. Cells were fixed for 20 min at RT, permeabilised for 15 min at RT, before staining for 1 h at 4°C in permeabilisation buffer.

Mitochondrial characteristics:
For mitochondria content and membrane potential, cells were incubated with MitoTracker™ Green FM (100 nM; M7514, ThermoFisher) or TMRE (50 nM; ab113852; Abcam) for 20 min at 37°C, respectively.

Reactive oxygen species
For mitochondrial ROS staining, cells were incubated with MitoSOX™ Red (5 µM; M36008, ThermoFisher) for 20 min at 37°C. For total ROS staining, cells were incubated with CellROX™ Green (5 µM; C10492, ThermoFisher).

Instrument

Human T cells were acquired on a Novocyte 3000 (Agilent).

Murine T cells were acquired on a Novocyte 3000 (Agilent), FACS Canto II or a Symphony A3 Cell Analyser (both BD).

Software

Data analysis was performed using FlowJo version 10 (TreeStar).

Cell population abundance

For isolated cells, purity was in excess of 90% analysed post autoMACS separation.

Gating strategy

Relevant cells were initially gated on FSC/SSC to exclude debris, then a gate identifying single cells was used (FSC-H v FSC-A). Cells were then stained with a target of interest whereby gating was determined using an unstained v stained sample. Gating strategies are present in the supplementary information.

☒ Tick this box to confirm that a figure exemplifying the gating strategy is provided in the Supplementary Information.

Magnetic resonance imaging

Experimental design

Design type

Indicate task or resting state; event-related or block design.

Design specifications

Specify the number of blocks, trials or experimental units per session and/or subject, and specify the length of each trial or block (if trials are blocked) and interval between trials.

Behavioral performance measures

State number and/or type of variables recorded (e.g. correct button press, response time) and what statistics were used to establish that the subjects were performing the task as expected (e.g. mean, range, and/or standard deviation across subjects).

Acquisition

Imaging type(s) | *Specify: functional, structural, diffusion, perfusion.*

Field strength | *Specify in Tesla*

Sequence & imaging parameters | *Specify the pulse sequence type (gradient echo, spin echo, etc.), imaging type (EPI, spiral, etc.), field of view, matrix size, slice thickness, orientation and TE/TR/flip angle.*

Area of acquisition | *State whether a whole brain scan was used OR define the area of acquisition, describing how the region was determined.*

Diffusion MRI ☐ Used ☒ Not used

Preprocessing

Preprocessing software | *Provide detail on software version and revision number and on specific parameters (model/functions, brain extraction, segmentation, smoothing kernel size, etc.).*

Normalization | *If data were normalized/standardized, describe the approach(es): specify linear or non-linear and define image types used for transformation OR indicate that data were not normalized and explain rationale for lack of normalization.*

Normalization template | *Describe the template used for normalization/transformation, specifying subject space or group standardized space (e.g. original Talairach, MNI305, ICBM152) OR indicate that the data were not normalized.*

Noise and artifact removal | *Describe your procedure(s) for artifact and structured noise removal, specifying motion parameters, tissue signals and physiological signals (heart rate, respiration).*

Volume censoring | *Define your software and/or method and criteria for volume censoring, and state the extent of such censoring.*

Statistical modeling & inference

Model type and settings | *Specify type (mass univariate, multivariate, RSA, predictive, etc.) and describe essential details of the model at the first and second levels (e.g. fixed, random or mixed effects; drift or auto-correlation).*

Effect(s) tested | *Define precise effect in terms of the task or stimulus conditions instead of psychological concepts and indicate whether ANOVA or factorial designs were used.*

Specify type of analysis: ☐ Whole brain ☐ ROI-based ☐ Both

Statistic type for inference | *Specify voxel-wise or cluster-wise and report all relevant parameters for cluster-wise methods.*

(See Eklund et al. 2016)

Correction | *Describe the type of correction and how it is obtained for multiple comparisons (e.g. FWE, FDR, permutation or Monte Carlo).*

Models & analysis

n/a	Involved in the study
☐	☐ Functional and/or effective connectivity
☐	☐ Graph analysis
☐	☐ Multivariate modeling or predictive analysis

Functional and/or effective connectivity | *Report the measures of dependence used and the model details (e.g. Pearson correlation, partial correlation, mutual information).*

Graph analysis | *Report the dependent variable and connectivity measure, specifying weighted graph or binarized graph, subject- or group-level, and the global and/or node summaries used (e.g. clustering coefficient, efficiency, etc.).*

Multivariate modeling and predictive analysis | *Specify independent variables, features extraction and dimension reduction, model, training and evaluation metrics.*
