# Supplementary material for: Mitochondrial ABHD11 inhibition drives sterol metabolism to modulate T-cell effector function
Source: Nat Commun. 2025 Nov 3;16:9484. doi: 10.1038/s41467-025-65417-4 (PMC12583646; doi:10.1038/s41467-025-65417-4)
Supplement: Supplementary file 5 — Source data 2 [file 41467_2025_65417_MOESM5_ESM.docx]

**SOURCE DATA 2**

**Title: Mitochondrial ABHD11 inhibition drives sterol metabolism to modulate T-cell effector function**

**Authors:** Benjamin J. Jenkins^1,†^, Yasmin R. Jenkins^1,†^, Fernando M. Ponce-Garcia^1^, Chloe Moscrop^2^, Iain A. Perry^1^, Matthew D. Hitchings^1^, Alejandro H. Uribe^3^, Federico Bernuzzi^3^, Simon Eastham^2^, James G. Cronin^1^, Ardena Berisha^4^, Alexandra Howell^5^, Joanne Davies^5^, Julianna Blagih^6,7^, Marta Williams^8^, Morgan Marsden^8^, Douglas J. Veale^9^, Luke C. Davies^1^, Micah Niphakis^10^, David K. Finlay^11^, Linda V. Sinclair^12^, Benjamin F. Cravatt^10^, Andrew E. Hogan^4^, James A. Nathan^13^, Ian R. Humphreys^8^, Ursula Fearon^14^, David Sumpton^3^, Johan Vande Voorde^3,15^, Goncalo Dias do Vale^16^, Jeffrey G. McDonald^16^, Gareth W. Jones^2^, James A. Pearson^5,‡^, Emma E. Vincent^17,18,‡^, Nicholas Jones^1,‡,*^

^1^ Institute of Life Science, Swansea University Medical School, Swansea University, SA2 8PP, UK.

^2^ Cellular and Molecular Medicine, University of Bristol, Biomedical Sciences Building, Bristol, BS8 1TD, UK.

^3^ Cancer Research UK Scotland Institute, Garscube Estate, Switchback Road, Glasgow, G61 1BD, UK.

^4^ Kathleen Lonsdale Institute for Human Health Research, Maynooth University, Maynooth, Co. Kildare, Ireland

^5^ Diabetes Research Group, Division of Infection and Immunity, School of Medicine, Cardiff University, CF14 4XN, UK

^6^ The Francis Crick Institute, 1 Midland Road, London, NW1 1AT, UK.

^7^ University of Montreal, Maisonneuve-Rosemont Hospital Research Centre, Montreal, 5414 Assomption Blvd, H1T 2M4, Canada

^8^ Division of Infection and Immunity/Systems Immunity University Research Institute, School of Medicine, Cardiff University, Cardiff, CF14 4XN, UK.

^9^ EULAR Centre of Excellence, Centre for Arthritis and Rheumatic Diseases, St Vincent’s University Hospital, Dublin, Ireland.

^10^ Department of Chemistry, Scripps Research, La Jolla, California 92037, United States

^11^ School of Biochemistry and Immunology, Trinity Biomedical Sciences Institute, Trinity College Dublin, 152-160 Pearce Street, Dublin, Ireland

^12^ Division of Cell Signalling and Immunology, School of Life Sciences, University of Dundee, Dundee, UK

^13^ Cambridge Institute of Therapeutic Immunology & Infectious Disease (CITIID), Jeffrey Cheah Biomedical Centre, Department of Medicine, University of Cambridge, Cambridge, CB2 0AW, UK.

^14^ Molecular Rheumatology, School of Medicine, Trinity Biomedical Sciences Institute, Trinity College Dublin, 152-160 Pearce Street, Dublin, Ireland.

^15^ School of Cancer Sciences, Wolfson Wohl Cancer Research Centre, University of Glasgow, Glasgow, G61 1QH, UK

^16^ Center for Human Nutrition, Department of Molecular Genetics, University of Texas Southwestern Medical Center, Dallas, United States.

^17^ School of Translational Health Sciences, Dorothy Hodgkin Building, University of Bristol, Bristol, BS1 3NY, UK.

^18^ Integrative Epidemiology Unit, School of Population Health Science, University of Bristol, Bristol, BS8 2BN, UK.

^†^ These authors contributed equally

^‡^ These authors jointly supervised this work

^*^ Corresponding author: Nicholas Jones, Institute of Life Science, Swansea University Medical School, Swansea, UK, SA2 8PP, +44 (0)1792 513509, n.jones@swansea.ac.uk

| 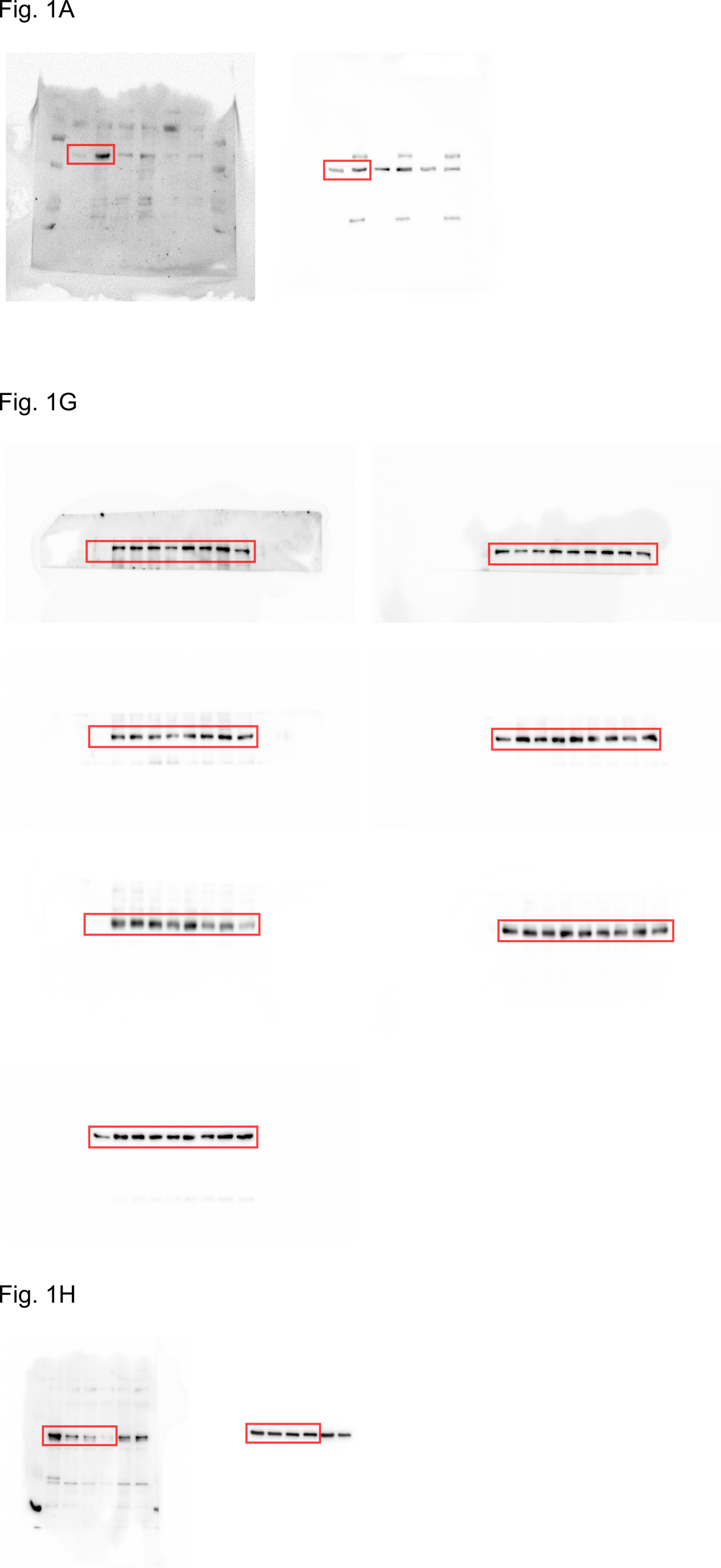 |
| --- |
